# Validation of a modified VOICES survey to measure end-of-life care quality: the CaregiverVoice survey

**DOI:** 10.1186/s12904-017-0227-7

**Published:** 2017-08-30

**Authors:** Hsien Seow, Daryl Bainbridge, Melissa Brouwers, Gregory Pond, John Cairney

**Affiliations:** 1 Department of Oncology, McMaster University, Hamilton, ON Canada; 2 Escarpment Cancer Research Institute, Hamilton, ON Canada; 3 Juravinski Cancer Centre, Hamilton, ON Canada; 4 Juravinski Hospital, Hamilton, ON Canada; 5 Kinesiology and Physical Education, University of Toronto, Toronto, ON Canada

**Keywords:** Palliative care, Measurement, Evaluation, Patient experience, Survey research, Quality of care, Outcomes, Bereaved caregivers

## Abstract

**Background:**

Measuring the care experience at end-of-life (EOL) to inform quality improvement is a priority in many countries. We validated the CaregiverVoice survey, a modified version of the VOICES questionnaire, completed by bereaved caregivers to capture perceptions of care received in the last three months of a patient’s life.

**Methods:**

We conducted a retrospective survey of bereaved caregivers representing palliative care patients who died in a residential hospice and/or received palliative homecare in Ontario, Canada. Statistical analyses were completed to establish construct and concurrent validity, as well as reliability of the survey.

**Results:**

Responses were obtained from 906 caregivers: 330 surveyed from homecare agencies and 576 from hospices. The CaregiverVoice survey demonstrated concurrent validity in scores correlating to FAMCARE2 items, and construct validity in performing according to expected patterns, e.g., correlation of scores to qualitative perceptions and significant variability based on care contexts such as place of death and setting of care (*p* < 0.01). Reliability was exhibited in good inter-item correlation of ratings for specific care settings and no significant differences in ratings regardless of whether up to a year had passed since death of patient.

**Conclusions:**

The CaregiverVoice survey demonstrated validity and reliability in the populations assessed. This survey represents one common measure that can be standardized across multiple care settings and is useful for assessing the care experience that can help inform local and national quality improvement activities.

**Electronic supplementary material:**

The online version of this article (doi:10.1186/s12904-017-0227-7) contains supplementary material, which is available to authorized users.

## Background

Patient reported outcomes consisting of patient/caregiver evaluations of care are critical to assessing end-of-life (EOL) services and targeting modifications or improvements, as needed [1, 2]. Many existing measures focus heavily on administrative data or on process data, as a surrogate for, but do not directly report on quality of the EOL care experience, particularly from the patient or caregiver perspective [3, 4]. As patients at the EOL often use multiple services from multiple settings, understanding the EOL experience beyond a single setting can provide insights into how to improve quality and better meet patient needs.

There are a diverse set of metrics that measure patient experience at the EOL. Many EOL care measures are short satisfaction type surveys. Examples such as FAMCARE [5] and the Palliative Outcome Scale [6] have been extensively used. While quick to administer, satisfaction scales typically focus on one setting of care, provide little context, and are prone to a ceiling effect [7, 8]. Alternatively, surveys that capture the care experience, rather than merely satisfaction, across multiple settings have a greater potential for identifying gaps in care that help to inform service quality improvement [9]. A systematic review identified 51 EOL experience measures, of which only 12 have been published on more than twice [10]. Of these, the majority focused on a single setting of care, such as an intensive care unit or residential hospice, and mostly during a period close to death (e.g., last week of life). Only one, the VOICES-SF (Views of Informal Carers Evaluation of Services Short Form) [11, 12] examined patient and family experience of EOL care in a comprehensive manner across different care settings, providers, and time points (i.e., the last 3 months of life). As such, the VOICES-SF has the potential to be used as a common survey instrument across all settings providing palliative care.

The VOICES-SF survey was endorsed by a provincial committee in Ontario, Canada, in 2012 to be used to measure patient and caregiver EOL experience. However, the survey was modified greatly for two main reasons. First, much of the language of the VOICES-SF survey is UK-centric, reflecting the country in which it was developed and most widely used. The survey needed to be adapted to Ontario to reflect the different health delivery system, different key provider stakeholders involved in palliative care, and emerging local policy priorities related to EOL. Second, a key purpose of the survey was to support organizations with quality improvement activities. As such, items that were deemed not actionable were reviewed, and items were added where more context was required. Since modifications were numerous, potentially altering key aspects of the VOICES-SF survey’s psychometric properties, they renamed the survey, the CaregiverVoice survey. The purpose of this study is to validate this modified questionnaire. The validation criteria were informed by the published guidelines of the Scientific Advisory Committee of the Medical Outcomes Trust (MOT) for assessing healthcare surveys [13].

## Methods

### CaregiverVoice survey development

The VOICES-SF survey is a commonly cited after-death follow-back questionnaire, completed by bereaved caregivers typically within a year of a patient’s death. This survey is unique in that it assesses quality in multiple settings where care was received in the last three months of life [10]. This instrument was used in the National Bereavement survey (2011 and 2012) in England [12, 14]. The VOICES-SF features a combination of question formats, including 4-point rating scales, multiple choice, and opened-ended items. The survey reviews different time periods, such as the last three months of life, the last two days of life, and circumstances surrounding the death. A series of questions target respect and dignity, pain management, and overall assessment of care in the setting. To allow for cross comparisons, the questions are repeated for each relevant setting: homecare, family physician care, urgent care provided out of hours, long-term care, last hospital admission, last residential hospice admission, experiences in the last week of life, and circumstances around death. The survey ends with two open-ended items asking the caregiver to describe what, if anything, was good about care, and bad about care, in the last three months of the patient’s life. The structure of the VOICES-SF enables several layers for comparison, including an overall rating score for care in the last 3 months of life, an overall rating for each specific setting, and ratings for particular domains of quality, such as pain management and respect and dignity.

The development of the CaregiverVoice survey has been described in detail elsewhere [15]. Briefly, using the VOICES-SF survey as foundation, engagement with target user groups of organizations, providers, and patient-family advisory councils, alignment with palliative care planning bodies, and review of relevant empirical literature on quality assessment, the following modifications were made:

- added sections related to hospice volunteer services, emergency department use, and cancer centre care
- added items about advance care planning and transitions in care
- added support domains of non-pain symptoms, emotional, and spiritual support in all settings of care (Hospice cohort survey version only)
- added fill-in-the-blank to include reason for particular events where more context was needed (e.g., reason for last ED visit, if applicable)
- revised support domains into a response array of five domains that is repeated for all settings of care, for ease of completion and to enable more systematic comparisons of care settings (Hospice cohort survey version only)
- revised time interval in items about the “last two days of life” to the “last week of life”
- revised homecare provider ratings to be separate for nurses and personal support workers
- revised a few response options that were previously identified as having issues of clarity and mutual exclusivity [16, 17]﻿;
- revised terminology to align with Canadian sample (e.g., from general practitioner to family doctor)
- deleted items that most respondents stated as not relevant during the pilot [15] or that were deemed not actionable by service providers and planners

The current version of the CaregiverVoice survey contains 62 items and takes approximately 20 min to complete. The survey can be completed on paper or online. Both versions contain skip logic so that caregivers only respond to items relevant to the types of care the patient received. Overall satisfaction with care in the last three months by specified setting is assessed on a four-point scale (1 = Excellent, 2 = Good, 3 = Fair, 4 = Poor).

### Study design and population

A retrospective, observational design was employed to test the survey with bereaved caregivers of decedents in Ontario, Canada who had received formal palliative care services in either the home or residential hospice. Unlike in the United States where hospice refers to specialized palliative care provided in any setting under the Medicare Hospice Benefits, in Canada and England hospice care denotes care that is provided in a residential hospice [18, 19]. In Ontario, homecare and hospice care are covered by a universally funded healthcare program. Palliative homecare is a special designation for patients residing at home and expected to live less than a year. They receive greater service entitlement of nursing or personal care workers, sometimes with specialized training [20].

The CaregiverVoice survey and the FAMCARE2 instrument [5] were administered together by 6 of 14 region-wide homecare provider organizations from September 2012 to January 2014 (homecare cohort) and by 16 of 25 residential hospices across the province from November 2014 to October 2015 (hospice cohort). Site selection was purposive in including those that agreed to participate in the study. The regions included represent both urban and rural areas. The CaregiverVoice survey underwent refinements between the homecare and hospice samples to improve comprehensiveness and ease of completion.

### Data collection

Family caregivers of deceased patients were identified through administrative review of client records by the participating organizations. The homecare sites employed a consecutive sampling method, contacting caregivers approximately six weeks after the patient died. Most hospice sites began with a retrospective approach, contacting caregivers of patients who had died in the past six months, and then from that point forward used the same prospective method as the homecare sites. In most cases, an initial contact was made to the caregiver to inform them of the survey and determine if an online or paper version of the survey was preferred. A paper survey or a letter with the survey link, as requested, were then mailed to the caregiver. Some sites made follow-up phone calls and mail contacts, often incorporated with bereavement support, to encourage survey completion.

Inclusion criterion for survey participation was the ability to read English, as this was the only language in which the survey was available. Completion of the survey was anonymous with no tracking of respondents. Additional details of the survey administration process were previously published [15].

### Data analysis

Data were imported into SPSS Version 23.0 (Armonk, NY: IBM Corp) for statistical computations. Descriptive statistics were used to summarize caregiver and patient characteristics and perceptions of services used. Validity and reliability analyses were conducted on the pooled sample on the homecare and hospice administered cohorts unless otherwise specified. All tests were two-sided and a *p*-value of 0.05 or less was considered statistically significant.

The MOT framework outlines eight attributes to critically reviewing healthcare surveys, [13] that were taken into account in validation of the CaregiverVoice survey. We tested for the following: (1) Conceptual and measurement model, i.e., empirical rationale for scale development; (2) Validity; (3) Reliability; (4) Respondent and administrative burden; and (5) Alternative forms of administration. We did not test for or extensively examine: (6) Responsiveness, i.e., detection of change over time; and (7) Cultural and language adaptations, due to limitations of the data and/or instrument. The eighth attribute, Interpretability, i.e., ease with which meaning can be derived from scores, was integral to the development of the CaregiverVoice Survey and data dissemination was guided by formal and informal input from the sites and other data users. However, we did not propose benchmarks or formally test the effectiveness of different knowledge translation approaches.

#### Conceptual and measurement model

Many of the criteria for assessing the conceptual soundness of the instrument overlap with the notion of face/content validity, that is, is there an empirical basis for item creation and do they appear to adequately capture the intended concept. The concept being, the quality of the end-of-life care experience. Also important is whether the dimensionality of the instrument corresponds to that of the intended concept or model. This aspect is covered under content validity.

#### Validity

**Criterion (concurrent) validity** is assessed by comparing how well the survey corresponds to a previously established measurement. In our study, caregivers in both samples concurrently completed the CaregiverVoice survey and the FAMCARE2 instrument [5]--which is considered a gold standard in measuring satisfaction with palliative care [21]. We used Spearman’s Rank Correlation to compare each respondent’s overall score on the CaregiverVoice survey to their overall (summary mean) score on the FAMCARE2 scale.

**Construct validity** attests as to whether the survey data conform to hypothesized patterns, in this case, of the palliative care experience among multiple settings. The scales adherence to expected patterns and intended dimensionality were tested in a number of ways. First, it was expected that caregivers’ good and/or bad open-ended (qualitative) comments about a specific care setting should correspond to their quantitative rating of that care setting. This was tested by quantifying qualitative comments that related tospecific settings into three groups, i.e., −1 = negative comment, 0 = no comment or both positive and negative comment, and 1 = positive comment. This coding was done independently by three analysts, with coding discrepancies discussed and resolved for complete agreement. Each respondent’s comment score for a care setting was compared to their scale rating of that setting using the Kruskal–Wallis test (hospice sample only due to the time required for qualifying the open text data).

Second, satisfaction with EOL care has been found to relate to place of death [16]. Most patients prefer not to die in a hospital institution, though the majority of deaths occur there, but rather in the home [22–25]. In Canada and elsewhere, hospice is the gold standard home-like setting [26]. Accordingly, we expected that a caregiver’s overall rating of care in the last 3 months of life would be most favorable for those who died in hospice, followed by homecare, and followed by hospital as least favorable. This hypothesis was tested using the Kruskal–Wallis test.

Third, we also expected that ratings within particular support domains to be different among settings. We expected each of the support domains within hospice, where specialized palliative care is available 24/7, to be higher ranked than homecare or hospital, and tested this using Wilcoxon signed-rank test for paired samples. This test was only done on the hospice cohort because few patients (18%) in the homecare cohort were cared for in hospice.

#### Reliability

**Internal consistency** analyses were done to test the consistency of the survey data across items within a construct. Cronbach’s alpha was calculated to determine the internal consistency between support domains (i.e., relief of pain, relief of other symptoms, spiritual support, and emotional support) within each given setting of care. To further test internal consistency of ratings within a care setting, a caregiver’s combined ratings of individual support domains for a specific care setting should correlate to their overall rating of care for that same setting. This was tested using Spearman’s Rank Correlation (Hospice sa﻿mple only due to specified survey item revisions).

**Reproductibility** is the survey’s consistency over time. It was not feasible to retest bereaved caregivers to determine the stability of the instrument by repeated measures. Rather, we assessed whether overall rating of care differed according to the length of time since the patient’s death, for which we used the Kruskal–Wallis test. Measurement error, and a threat to reliability, would be evident if caregivers who respond closer to the patient’s death (e.g., 2 months later) are systematically different than those who respond longer after a patient’s death (e.g., 9 months later) [7].

#### Respondent and administrative burden

Measures used to assess the burden of the survey are the mean completion time, reading comprehension level, and percent of missing data. Other potential barriers for respondents and administrators are discussed.

#### Alternative forms of administration

We compared caregiver’s overall ratings of care between paper and on-line versions of the CaregiverVoice survey using the Kruskal–Wallis test. The effect of different methods of survey administration on response rates was previously reported [15].

## Results

### Demographics

Responses were obtained from 906 caregivers: 330 from homecare and 576 from hospice populations. Deceased patient characteristics are presented in Table 1. Regardless of the site of survey administration or place of death, most patients received care from multiple settings in the last three months of life. The main settings of care indicated in the last three months of life were home (81%), hospital (56%), and residential hospice (70%), with 51% of patients using at least two of these main settings. Half of patients were male and 67% were 70 years or older. The major illness reported for most patients was cancer (81%). The caregiver respondents tended to be younger than patients and female (69%). Just under a third of caregivers were the patient’s son or daughter and 54% were the patient’s spouse. Responses showed 23% died at home, 9% in hospital, and 67% in hospice, and 1% in a long-term care home. Caregivers’ responses to the overall rating of care in the patient’s last 3 months of life were positively skewed, 31% indicating the best category (excellent care [“1”]) and 2% indicating the worst category (poor care [“5”]). Almost all respondents (95%) provided open-text comments describing what was good or bad about care.

**Table 1:** Demographics of deceased patients (*n* = 906)

	All	Hospice Cohort (*n* = 576)	Homecare Cohort (*n* = 330)
Sex	N (%)	N (%)	N (%)
Male	453 (50.8)	298 (52.8)	155 (47.3)
Female	439 (49.2)	266 (47.2)	173 (52.7)
Age			
Under 50	29 (3.2)	13 (2.3)	16 (4.9)
50–69	268 (29.8)	158 (27.7)	110 (33.4)
70–89	505 (56.2)	333 (58.4)	172 (52.3)
90+	97 (10.8)	66 (11.6)	31 (9.4)
Main Diagnosis			
Cancer	730 (80.6)	459 (79.7)	271 (82.1)
Kidney or Liver Disease	30 (3.3)	23 (4.0)	7 (2.1)
Heart Disease	28 (3.1)	19 (3.3)	9 (2.7)
Alzheimer’s or other Neurological Diseases	22 (2.4)	13 (2.3)	9 (2.7)
COPD/Asthma	16 (1.8)	7 (1.2)	9 (2.7)
Stroke	13 (1.4)	13 (2.3)	(0.0)
Other or Unknown	67 (7.4)	42 (7.3)	25 (7.6)
Settings/Providers of Care^a^			
Received homecare	730 (80.6)	400 (69.4)	330 (100.0)
Had visiting hospice volunteers	135 (14.9)	74 (12.8)	61 (18.5)
Stayed in hospital	504 (55.6)	316 (54.9)	188 (57.0)
Stayed in residential hospice	636 (70.2)	576 (100.0)	60 (18.2)
Stayed in long term care home	88 (9.7)	64 (11.1)	24 (7.3)
Visited emergency department^b^		338 (58.7)	
Palliative care doctor was MRP	422 (46.6)	255 (44.3)	167 (50.6)

^a^In the last three months of life – multiple settings and providers are possible

^b^emergency department use only asked in Hospice cohort

MRP = Most Responsible Physician

Percentages exclude missing data

### Assessment of survey validity

#### Face validity

We do not report extensively on the recognized face/content validity of the CaregiverVoice survey for the sake of brevity. The VOICES-SF that served as a basis for the survey has been extensively tested and reviewed for face validity by experts [12, 17]. Furthermore, throughout the refinement process, the CaregiverVoice survey items and data generated were vetted with a wide scope of caregivers, providers, administrators, and survey methodologists, as previously described. This survey covers the majority of the domains present in established palliative care theoretical frameworks, including the domains outlined by the National Consensus Project for Quality Palliative Care [27].

#### Criterion (concurrent) validity

A significant association was found between caregivers’ overall rating of care on the CaregiverVoice survey and their summary score on the FAMCARE2 items, (*n* = 855, r_s_ = 0.66), *p* < 0.001.

#### Construct validity

First, the associations between caregivers’ care-setting specified “good” and/or “bad” qualitative comments and the respective quantitative rating were analyzed for the three mostly commonly accessed care sites: homecare, hospital, and hospice. Among the hospice sample examined, there were significant relationships in the appropriate direction for each of homecare (*n* = 390, Kruskal–Wallis H = 28.9), hospital (*n* = 310, Kruskal–Wallis H = 53.9), and hospice (*n* = 523, Kruskal–Wallis H = 50.9), all *p* < 0.001. That is, higher qualitative coded scores were directly associated with higher scale scores, for each respective care setting. This indicates that the overall rating scales for these settings appropriately capture caregivers’ expressed perceptions of the care experience.

Second, caregivers’ ratings of overall satisfaction with patient care in the last three months of life varied significantly depending on the place of death reported: hospice Mean = 1.99 [34% rated excellent], home Mean = 2.11 [27% rated excellent], and hospital Mean = 2.34 [16% rated excellent] (range 1 = excellent to 4 = poor). Hospital place of death corresponded to a lower overall satisfaction rating than home, and home corresponded to lower overall satisfaction rating than hospice (*n* = 847, Kruskal–Wallis H = 10.9), *p* = 0.004.

Third, each caregiver’s ratings of the four support domains differed between hospice, homecare, and hospital. As expected, hospice ratings were more positive than homecare or hospital as seen in Table 2. For instance, significant differences in the domain ratings between hospice and homecare were as follows: Relief of physical pain (*n* = 312, Z = 275), Relief of other symptoms (*n* = 306, Z = 507), Spiritual support (*n* = 157, Z = 308), and Emotional support (*n* = 226, Z = 558), all *p* < 0.001, with hospice consistently perceived as more supportive than homecare.

**Table 2:** Caregiver support domain ratings of select settings of care in last 3 months of life (*n* = 576) (hospice cohort only)

Support domain assessed^a^	Hospice		Homecare		Last hospital admission		Site rating comparisons	
	N	Mean (SD)	N	Mean (SD)	N	Mean (SD)	Hospice– Homecare *p-*value^b^	Hospice– Hospital *p-*value^b^
Relief of physical pain	511	1.19 (0.51)	344	1.97 (0.88)	297	1.88 (0.91)	<0.001	<0.001
Relief of other symptoms	497	1.24 (0.53)	343	2.06 (0.83)	282	2.01 (0.91)	<0.001	<0.001
Spiritual support	345	1.28 (0.61)	182	2.14 (0.95)	167	2.14 (1.07)	<0.001	<0.001
Emotional support	466	1.20 (0.49)	308	2.06 (0.89)	248	2.24 (1.02)	<0.001	<0.001
Overall rating	523	1.08 (0.32)	390	1.76 (0.79)	310	1.80 (0.80)	<0.001	<0.001

^a^1 to 4 range, scale options 1 = Excellent, 2 = Good, 3 = Fair, 4 = Poor

^b^pair wise comparisons using Wilcoxon signed-rank test for paired samples

### Assessment of survey reliability

#### Internal consistency

Good internal consistency was found between individual’s ratings of each support domain within the given care settings. For example, the internal consistency between ratings of Relief of physical pain, Relief of other symptoms, Spiritual support, and Emotional support for homecare, hospital, and hospice were respectively: Cronbach’s α = 0.84 (*n* = 165), α = 0.93 (*n* = 155), and α = 0.81 (*n* = 334). Furthermore, individual’s combined (summary mean) ratings of the support domains for each of the main care settings correlated strongly to their overall rating of care for that setting. For example, home (*n* = 347, r_s_ = 0.73), hospital (*n* = 292, r_s_ = 0.70), and hospice (*n* = 504, r_s_ = 0.59), all *p* < 0.001.

#### Reproductibility

The majority (37%) of patients represented were reported at survey completion to have died from 6 months to a year ago, however the range was dispersed, from less than 2 months (14%) to over a year (7%). No significant differences were detected in overall rating of care regardless of time since patient’s death to survey completion (*n* = 547, Kruskal–Wallis H = 8.7), *p* = 0.07.

### Assessment of respondent and administrative burden

The CaregiverVoice survey is intended to take 15 to 30 min to complete depending on the number of care settings rated and the open-ended comments provided. The actual median completion time for the online version of survey was 25.7 min, with an interquartile range of 18.7 min. There was less than 5% missing data and 97.1% of respondents completed the whole survey. The Flesch-Kincaid grade level is 8.5 and reading ease score is 65.1, indicating that the survey is in plain English, even with the use of more advanced terminology such as “advance care planning” [28]. As mentioned, the majority of caregivers provided open-text comments, further suggesting that the quantitative portion of the survey is not overly burdensome.

The option to complete the survey on-line greatly reduces the administrative burden by eliminating the time and cost of printing, mailing, and data entry. Speaking with the caregiver (e.g., phone contact) is critical to ensuring a reasonable response rate [15]. Some of the hospices used volunteers to contact caregivers about the survey, however, many of the sites expressed challenges in re-allocating limited resources to administer the survey.

### Assessment of alternative forms of administration

Nearly two-thirds (65.3%) of respondents completed the survey online, rather than on paper. No significant differences were detected in overall rating of care regardless of the mode of completion (*n* = 868, Kruskal–Wallis H = 0.004), *p* = 0.95.

## Discussion

This study investigated the validity and reliability of the CaregiverVoice survey, an adapted and heavily-modified version of the UK’s VOICES survey, among a large cohort of over 900 patients, most of whom died in hospice or at home. The CaregiverVoice survey incorporated additions to cover the recognized key domains of quality palliative care including emotional and spiritual support, as well as, advance care planning and transitions between care settings. Furthermore, revisions were made to the survey to help ensure that the data produced provides actionable insight for the quality improvement activities of service providers and planners.

Our validation of the CaregiverVoice survey included several tests of concurrent validity, demonstrating variability and results in the expected directions. The survey also demonstrated high internal consistency among support domains. Concurrent validity was also evident against the gold standard FAMCARE2, which is the most widely used end-of-life satisfaction tool. Small differences in the global rating scores between the two instruments may be explained by the different response options (satisfaction vs experience) and scope of consideration (major setting of care vs all settings), for the FAMCARE2 and CaregiverVoice surveys, respectively.

Overall, the survey performed well according to the MOT framework criteria we measured. The CaregiverVoice survey is useful for helping to measure and assess quality (via the overall scores), but also informing local activities to improve the quality of EOL care (via open ended questions and contextual comments). Further validation work is required to determine instrument Responsiveness, to assess the survey’s sensitivity to changes in care provision. Non-English language adaptations of the CaregiverVoice survey also need to be investigated to increase inclusivity of the data.

The importance of the CaregiverVoice survey resides in that it represents one common survey that can be standardized across multiple settings that provide EOL care. The survey spans diverse care settings and providers across the last 3 months of life, including circumstances surrounding and after death, allowing it to address the many transition points in the last months of life. Even providers who focus primarily on curative instead of palliative care can benefit from feedback obtained through the survey. Indeed, data in Ontario shows the majority of palliative care services occur less than two months from death [29]. Thus helping providers to incorporate palliative and EOL care earlier in the disease trajectory is a major area for improvement that the survey could help highlight. To truly inform quality improvement activities and improve care, logistical issues such as data ownership, data reporting, data collection and sampling, and sharing of results across settings will need to be addressed.

Limitations in the validation process include that we were unable to conduct test-retest analysis, due to the survey being anonymous and sensitivities in asking bereaved caregivers to complete the survey twice. However, a test-retest analysis was previously done on the VOICES survey (3–5 months compared to 7–9 months) that demonstrated a considerable to moderate degree of consistency found in ratings of management of pain and anxiety [30]. A limitation of our sample is that it was not representative of all patients at EOL, only those who utilized specialized EOL care services. We did not survey caregivers of patients who died in hospital or nursing homes, although use of these care settings is represented. Our current sample size is large, diverse, and statistically sufficient for the validation tests. As previously noted, we were not able to include the homecare cohort data in some of the tests due to item changes between these versions of the survey. To help normalize the response distribution, the core item response options in the next iteration of the CaregiverVoice survey will be changed from 4-point rating scales to 5-point scales (1 = Excellent, 2 = Very Good, 3 = Good, 4 = Fair, 5 = Poor), which research has shown improves questionnaire reliability [7] and initial testing of the survey has found reduced response skewness. Finally, some of the care health care provider nomenclature used in the questionnaire is specific to the health care system in Ontario or Canada (e.g., family physician); however, the principles of quality care assessed are global. The professional roles of providers are also universal in palliative care [31–33] and the nomenclature can be easily modified for relevance to the system and language assessed. These changes to the survey would likely retain the psychometric properties of the version we tested, as long as the content assessed remains the same. Future directions are to use the survey in broader, more representative, and culturally diverse populations. A study of a large representative homecare sample in Ontario is underway using the survey in multiple languages. Additionally, a population-based representative sample is being explored as a next research study that would include a larger proportion of long-term care and hospital populations.

## Conclusions

This study validated the CaregiverVoice survey using the Medical Outcomes Trust framework. Results demonstrate that key psychometric properties of the VOICES survey, from which it was adapted, were maintained. The uniformity (i.e., repeating of core items) of the CaregiverVoice survey makes it useful for comparing ratings of care between settings and across regions. This survey represents one common measure that can be standardized across multiple care settings, which can inform local and national quality improvement activities to improve care experience. The “Interpretability” of the data from the instrument requires further consideration, particularly the refinement of knowledge translation strategies to assist the care settings measured in using these “patient” reported outcomes towards quality improvement.

## Additional file

Additional file 1: CaregiverVoice survey. (PDF 386 kb)

## Abbreviations

EOL: End-of-life
MOT: Medical Outcomes Trust
MRP: Most Responsible Physician
VOICES-SF: Views of Informal Carers Evaluation of Services Short Form
