# Supplementary material for: Validation of a modified VOICES survey to measure end-of-life care quality: the CaregiverVoice survey
Source: BMC Palliat Care. 2017 Aug 30;16:44. doi: 10.1186/s12904-017-0227-7 (PMC5577821; doi:10.1186/s12904-017-0227-7)
Supplement: Additional file 1: — CaregiverVoice survey. (PDF 386 kb) [file 12904_2017_227_MOESM1_ESM.pdf]

Survey ID: __ __ __ __ - __ __ __ __

CaregiverVoice Survey

This survey is about the experiences of care and the services received by you and your family member/friend in the last months of his/her life. The information you give will help us improve care for people who are dying, and for their family and friends. Your views are, therefore, important to us. We realize this questionnaire may bring back strong memories.

Some of the questions may not be relevant to you. Please fill in as much of the survey as you can, or if you would rather not answer one of the questions, please go on to the next one.

Instructions: As you go through the survey, please follow the instructions and answer the questions by ticking the most appropriate box or boxes, like this ☑. Tick only one answer to each question unless the question states otherwise.

The survey takes about 20 minutes to complete.

Please complete the survey at your earliest convenience and return the completed survey in the stamped, self-addressed envelope provided.

PART A: Family Member/Friend Information

The first set of questions asks some general information about the family member/friend you cared for.

1. What was the gender of this person?

❑ Male ❑ Female ❑ Transgender/Other

2. How old was he/she when he/she died?

❑ 0-17 ❑ 50-69 ❑ 90+
❑ 18-29 ❑ 70-89 ❑ Don't know
❑ 30-49

3. How long ago did he/she die?

❑ Less than 2 months ago ❑ 6 months to 1 year ago
❑ 2 to 4 months ago ❑ Longer than 1 year ago
❑ 4 to 6 months ago

4. What was his/her **main** illness in the **last 3 months** of life? *Tick one only*

❑ Cancer ❑ Influenza or Pneumonia
❑ Heart Disease, e.g., congestive heart failure ❑ Neurological Disease, e.g., Alzheimer's, ALS
❑ Stroke ❑ Kidney or Liver Disease
❑ Lung Disease, e.g., COPD, asthma ❑ Other
❑ Diabetes ❑ Don't know

5. Did he/she identify him/herself as Aboriginal? (e.g., North American Indian, First Nations, Métis, or Inuit)

❑ Yes ❑ No **– go to Q6** ❑ Don't know **– go to Q6**
⇩
If YES, which were they? *Tick one only*

❑ First Nation (status) ❑ Métis ❑ Don't know
❑ First Nation (non-status) ❑ Inuk ❑ Other, please specify: ________________

Was his/her permanent address on a First Nations territory or reserve?

❑ Yes ❑ No

6. What was his/her religion? *Tick one only*

- ❑ No religion
- ❑ Christian (all denominations)
- ❑ Buddhist
- ❑ Hindu
- ❑ Jewish
- ❑ Muslim
- ❑ Sikh
- ❑ Other
- ❑ Don't Know

The next set of questions asks some general information about you.

7. What was your relationship to him/her? You were the: *Tick one only*

- ❑ Married spouse
- ❑ Common law spouse
- ❑ Son/daughter
- ❑ Brother/sister
- ❑ Brother-in-law/sister-in-law
- ❑ Parent/parent-in-law
- ❑ Son/daughter-in-law
- ❑ Other

8. What is your gender?

- ❑ Male
- ❑ Female
- ❑ Transgender/Other

9. How old are you?

- ❑ 16-29
- ❑ 30-49
- ❑ 50-69
- ❑ 70-89
- ❑ 90+

<u>PART B</u>: Care in Different Settings

The questions in the next sections are about care your family member/friend may have received in different settings, as specified.

<u>Homecare</u>

10. Did he/she have care from **<u>homecare providers</u>** (nurses, personal support workers, social workers, etc.) contracted by the Community Care Access Centre (CCAC) in the **<u>last 3 months</u>** of life?

- ❑ Yes
- ❑ No – **go to Q15**

11. When he/she was at home in the **last 3 months** of life, did the **homecare providers** work well together?

- ❑ Yes, definitely
- ❑ Yes, to some extent
- ❑ No, they did not work well together
- ❑ Don't know

12. Overall, do you feel that you and your family got as much help and support from **homecare services** as you needed?

- ❑ Yes, we got as much support as we needed
- ❑ No, we did not get as much support as we needed though we tried to get more
- ❑ No, we did not get as much support as we needed, but we did not ask for more

13. During the **last 3 months** of his/her life, while he/she was receiving **homecare services**, what is your assessment of the overall level of support given in the following areas?

	Excellent	Very Good	Good	Fair	Poor	Does not Apply	Don't Know
Relief of physical pain	❑	❑	❑	❑	❑	❑	❑
Relief of other symptoms	❑	❑	❑	❑	❑	❑	❑
Spiritual support	❑	❑	❑	❑	❑	❑	❑
Emotional support	❑	❑	❑	❑	❑	❑	❑
Practical support with activities of daily living	❑	❑	❑	❑	❑	❑	❑
Respect and dignity	❑	❑	❑	❑	❑	❑	❑

14. Overall, do you feel that the care he/she got from the nurses, personal support workers (PSW's), and CCAC care coordinators in the **last 3 months** of life was: *Answer individually for each*

	Excellent	Very Good	Good	Fair	Poor	Does not Apply	Don't Know
Nurses	❑	❑	❑	❑	❑	❑	❑
Personal Support Workers (PSW's)	❑	❑	❑	❑	❑	❑	❑
CCAC Care Coordinators	❑	❑	❑	❑	❑	❑	❑

Care from a Clinician (i.e., Family Doctor, Other Doctor, or Nurse Practitioner)

15. In the **last 3 months** of his/her life, which type of clinician provided the most of his/her care? *Tick one only*

- ❑ His/her family doctor
- ❑ Palliative care doctor
- ❑ Nurse practitioner
- ❑ Oncologist – **go to Q17**
- ❑ Other doctor/specialist (e.g., cardiologist) – **go to Q17**
- ❑ He/she never saw a clinician – **go to Q19**
- ❑ Don't know – **go to Q19**

16. Did the **clinician you selected above** visit him/her **at home** in the **last 3 months** of life?

❑ Yes ❑ No ❑ Don't know

⇩ **If Yes**, how easy or difficult it was to get this home visit? *Tick one only*

- ❑ Very easy
- ❑ Fairly easy
- ❑ Fairly difficult
- ❑ Very difficult
- ❑ Don't know

⇩ **If No**, why not? *Tick one only*

- ❑ He/she did not need a home visit by a clinician
- ❑ He/she needed a home visit by a clinician but could not get a visit
- ❑ Don't know

17. During the **last 3 months** of his/her life, while he/she was being cared for by the **clinician you selected above** (Question 15), what is your assessment of the overall level of support given in the following areas?

	Excellent	Very Good	Good	Fair	Poor	Does not Apply	Don't Know
Relief of physical pain	❑	❑	❑	❑	❑	❑	❑
Relief of other symptoms	❑	❑	❑	❑	❑	❑	❑
Spiritual support	❑	❑	❑	❑	❑	❑	❑
Emotional support	❑	❑	❑	❑	❑	❑	❑
Respect and dignity	❑	❑	❑	❑	❑	❑	❑

18. Overall, do you feel that the care he/she got from this **clinician** in the **last 3 months** of life was:

❑	❑	❑	❑	❑	❑
Excellent	Very Good	Good	Fair	Poor	Don't know

Urgent Care Provided Out of Normal Business Hours

19. In the **last 3 months** of life, while he/she was at **home**, how often did he/she ever need to contact a health professional for something **urgent** in the evening or during the weekend?

- ❑ Not at all in the last 3 months – **go to Q22**
- ❑ Once or twice
- ❑ Three or four times
- ❑ Five times or more
- ❑ Not applicable – **go to Q22**
- ❑ Don't know – **go to Q22**

20. The **last time this happened**, who did he/she contact, or who was contacted on his/her behalf? *Tick one only*

- ❑ His/her family doctor or the doctor's after-hours number
- ❑ His/her palliative care doctor
- ❑ Homecare provider
- ❑ Nurse practitioner
- ❑ Both a doctor and a homecare provider
- ❑ Hospice
- ❑ 911 or used lifeline pendant
- ❑ Other

21. What happened as a result? *Tick one only*

- ❑ Visited by his/her family doctor at home
- ❑ Visited by a palliative care doctor or another doctor at home
- ❑ Visited by a homecare provider at home
- ❑ Visited by both a doctor and a homecare provider
- ❑ Visited by a nurse practitioner at home
- ❑ Given medical advice over the telephone
- ❑ Given another number to call to get medical advice
- ❑ Advised to call 911
- ❑ Advised to go to an Emergency Department at a hospital
- ❑ Other

Visiting Hospice Volunteers

22. Did he/she get help from **visiting hospice volunteer(s)** in the home in the **last 3 months** of life?

- ❑ Yes
- ❑ No – **go to Q24**
- ❑ Don't know – **go to Q24**

23. Indicate your opinion about the help he/she got from the **visiting hospice volunteer(s)** in the **last 3 months** of life as to the following:

	Strongly Agree	Agree	Neither	Disagree	Strongly Disagree	Does not Apply	Don't Know
a) Helped to improve his/her wellbeing	❑	❑	❑	❑	❑	❑	❑
b) Allowed me to take time for myself, which contributed to my wellbeing	❑	❑	❑	❑	❑	❑	❑

Cancer Centre

24. Did he/she receive care at a **cancer centre** during his/her **last 3 months** of life?

❑ Yes, please name the cancer centre he/she spent the most time in: ___________

____

❑ No – **go to Q27**

❑ Don't know – **go to Q27**

25. During the **last 3 months** of his/her life, when he/she was being cared for by the **cancer centre** you named, what is your assessment of the overall level of support given in the following areas?

	Excellent	Very Good	Good	Fair	Poor	Does not Apply	Don't Know
Relief of physical pain	❑	❑	❑	❑	❑	❑	❑
Relief of other symptoms	❑	❑	❑	❑	❑	❑	❑
Spiritual support	❑	❑	❑	❑	❑	❑	❑
Emotional support	❑	❑	❑	❑	❑	❑	❑
Respect and dignity	❑	❑	❑	❑	❑	❑	❑

26. Overall, do you feel that the care he/she got from the **cancer centre** you named, in the **last 3 months** of life was:

❑	❑	❑	❑	❑	❑
Excellent	Very Good	Good	Fair	Poor	Don't know

Long Term Care Homes

27. Did he/she live or stay in a **Long Term Care home** at any time during his/her **last 3 months** of life?

- ❑ Yes, please name: ____
- ❑ No – **go to Q30**
- ❑ Don't know – **go to Q30**

28. During the **last 3 months** of his/her life, while he/she was in a **Long Term Care home**, what is your assessment of the overall level of support given in the following areas?

	Excellent	Very Good	Good	Fair	Poor	Does not Apply	Don't Know
Relief of physical pain	❑	❑	❑	❑	❑	❑	❑
Relief of other symptoms	❑	❑	❑	❑	❑	❑	❑
Spiritual support	❑	❑	❑	❑	❑	❑	❑
Emotional support	❑	❑	❑	❑	❑	❑	❑
Respect and dignity	❑	❑	❑	❑	❑	❑	❑

29. Overall, do you feel that the care he/she got from the **Long Term Care home** in the **last 3 months** of life was:

❑	❑	❑	❑	❑	❑
Excellent	Very Good	Good	Fair	Poor	Don't know

Last Hospital Admission

30. How often in the **last 3 months** of life did he/she have to go to the **Emergency Department (ED)**?

- ❑ Not at all **– go to Q32**
- ❑ Once or twice
- ❑ Three or four times
- ❑ Five times or more
- ❑ Don't know **– go to Q32**

31. What was the **main** reason for his/her **last Emergency Department (ED)** visit? *Tick one only*

- ❑ Altered consciousness or fatigue
- ❑ Pain issues
- ❑ Other symptom issues (e.g., nausea, shortness of breath)
- ❑ Infection
- ❑ Caregiver respite/unable to manage at home
- ❑ Multiple issues
- ❑ Other reason
- ❑ Don't know

32. How many days **total** did he/she stay in **hospital**, including a palliative care unit, during his/her **last 3 months** of life?

- ❑ None at all **– go to Q36**
- ❑ Less than 7 days
- ❑ 7 to 13 days
- ❑ 2 weeks to 4 weeks
- ❑ more than 4 weeks
- ❑ Don't know **– go to Q36**

33. What was the **main** reason for his/her **last hospital admission**: *Tick one only*

- ❑ Treatment of disease (e.g., chemo, surgery, tests, follow up)
- ❑ Pain issues
- ❑ Other symptom issues (e.g., nausea, shortness of breath)
- ❑ Infection
- ❑ Caregiver respite/unable to manage at home
- ❑ Multiple issues
- ❑ Other reason
- ❑ Don't know

Name of **hospital**: ____

34. During his/her **last hospital admission**, what is your assessment of the overall level of support given in the following areas?

	Excellent	Very Good	Good	Fair	Poor	Does not Apply	Don't Know
Relief of physical pain	❑	❑	❑	❑	❑	❑	❑
Relief of other symptoms	❑	❑	❑	❑	❑	❑	❑
Spiritual support	❑	❑	❑	❑	❑	❑	❑
Emotional support	❑	❑	❑	❑	❑	❑	❑
Respect and dignity	❑	❑	❑	❑	❑	❑	❑

35. Overall, do you feel that the care he/she got from the **hospital** on that admission was:

❑	❑	❑	❑	❑	❑
Excellent	Very Good	Good	Fair	Poor	Don't Know

Residential Hospice Admission

36. Did he/she stay in a **hospice** at any time during his/her **last 3 months** of life?

- ❑ Yes, please name: ____
- ❑ No – **go to Q39**
- ❑ Don't know – **go to Q39**

37. During the **last 3 months** of his/her life, while he/she was in **hospice**, what is your assessment of the overall level of support given in the following areas?

	Excellent	Very Good	Good	Fair	Poor	Does not Apply	Don't Know
Relief of physical pain	❑	❑	❑	❑	❑	❑	❑
Relief of other symptoms	❑	❑	❑	❑	❑	❑	❑
Spiritual support	❑	❑	❑	❑	❑	❑	❑
Emotional support	❑	❑	❑	❑	❑	❑	❑
Respect and dignity	❑	❑	❑	❑	❑	❑	❑

38. Overall, do you feel that the care he/she got from the **hospice** was:

❑	❑	❑	❑	❑	❑
Excellent	Very Good	Good	Fair	Poor	Don't Know

PART C: Overall Care

39. Overall, and taking all services, in all settings into account, how would you rate his/her care in the **last 3 months** of life?

❑	❑	❑	❑	❑	❑
Excellent	Very Good	Good	Fair	Poor	Don't Know

PART D: Experiences in the Last Week of Life

40. Where did your family member/friend receive most of their care in the **last week of life**: (if equal time was spent in 2 or more settings, select the one most used towards the end of their life) *Tick one only*

- ❑ Home with Homecare services
- ❑ Home without Homecare services
- ❑ Long term care home
- ❑ Retirement home
- ❑ Residential hospice
- ❑ Hospital acute or intensive care
- ❑ Hospital complex continuing care unit (CCC Unit)
- ❑ Hospital palliative care unit (PCU)
- ❑ Other

41. Thinking about the **setting you selected above** (Question 40), indicate your opinion about the help he/she received in the **last week of life** to each of the following:

	Strongly Agree	Agree	Neither	Disagree	Strongly Disagree	Does not Apply	Don't Know
a) There was enough help available to meet his/her personal care needs (such as toileting needs)	❑	❑	❑	❑	❑	❑	❑
b) There was enough help with nursing care, such as giving medicine or helping him/her find a comfortable position in bed	❑	❑	❑	❑	❑	❑	❑
c) The bed area and surrounding environment had adequate privacy for him/her	❑	❑	❑	❑	❑	❑	❑
d) There was enough support to stay where he/she wanted to be	❑	❑	❑	❑	❑	❑	❑

42. During the **<u>last week of life for the setting you specified</u>** (Question 40) what is your assessment of the level of support given in the following areas?

	Excellent	Very Good	Good	Fair	Poor	Does not Apply	Don't Know
Relief of physical pain	❑	❑	❑	❑	❑	❑	❑
Relief of other symptoms	❑	❑	❑	❑	❑	❑	❑
Spiritual support	❑	❑	❑	❑	❑	❑	❑
Emotional support	❑	❑	❑	❑	❑	❑	❑
Respect and dignity	❑	❑	❑	❑	❑	❑	❑

<u>PART E</u>: Transitions

43. To what extent did he/she experience <u>smooth transitions</u> between all settings of care during the **<u>last 3 months</u>** of life?

❑ Always **– go to Q46**
❑ Most of the time
❑ Sometimes
❑ Rarely
❑ Never
❑ Don't know **– go to Q46**

44. Identify the <u>worst</u> transition he/she experienced by checking the two settings where this transition began and finished. Only record the <u>worst</u> transition experienced.

Setting he/she started in:	Setting he/she transitioned to:
❑ Home <u>with</u> Homecare services	❑ Home <u>with</u> Homecare services
❑ Home <u>without</u> Homecare services	❑ Home <u>without</u> Homecare services
❑ Hospital bed	❑ Hospital bed
❑ Emergency department or Urgent care clinic	❑ Emergency department or Urgent care clinic
❑ Long term care home	❑ Long term care home
❑ Retirement home	❑ Retirement home
❑ Hospice	❑ Hospice

45. What made this transition so poor?

____

____

____

<u>PART F</u>: Circumstances Surrounding His/Her Death

46. Did he/she know he/she was likely to die?

- ❑ Yes, certainly
- ❑ Yes, probably
- ❑ Probably not
- ❑ No, definitely not
- ❑ Not sure

47. How long had he/she been ill before he/she died? *Tick one only*

- ❑ He/she was not ill – he/she died suddenly
- ❑ Less than 24 hours
- ❑ One day or more, but less than one week
- ❑ One week or more, but less than one month
- ❑ One month or more, but less than six months
- ❑ Six months or more, but less than one year
- ❑ One year or more

48. Where did he/she die? *Tick one only*

- ❑ At home
- ❑ In a hospice
- ❑ In a long term care home
- ❑ In a retirement home
- ❑ In a hospital acute or intensive care unit or emergency department
- ❑ In a hospital palliative care unit (PCU)
- ❑ In a hospital complex continuing care unit (CCCU)
- ❑ Other

49. Did he/she ever say where he/she would like to die?

- ❑ Yes
- ❑ No – **go to Q52**
- ❑ Not sure – **go to Q52**

50. What was his/her **<u>last known</u>** preferred place to die? *Tick one only*

- ❑ At home
- ❑ In a hospice
- ❑ In a hospital
- ❑ In a long term care home
- ❑ In a retirement home
- ❑ He/she said that he/she did not mind where he/she died
- ❑ Anywhere except at home
- ❑ Anywhere except in hospital
- ❑ Don’t know – go to Q52

51. Did the health care providers have a record of this preference of where he/she wanted to die?

- ❑ Yes
- ❑ No
- ❑ Not sure

52. Do you think that he/she died in the right place?

- ❑ Yes
- ❑ No
- ❑ Not sure

53. Were you or his/her family given enough help and support by the health care providers at the actual time of his/her death?

- ❑ Yes, definitely
- ❑ Yes, to some extent
- ❑ No, not at all
- ❑ Don't know

54. Looking back over the **last 3 month**s of his/her life, was he/she involved in decisions about his/her care as much as he/she would have wanted? *Tick one only*

- ❑ He/she was involved as much as he/she wanted to be
- ❑ He/she would have liked to be more involved
- ❑ He/she would have liked to be less involved
- ❑ He/she wasn't able to be involved due to incapacity
- ❑ Don't know

55. Looking back over the **last 3 months** of his/her life, were you involved in decisions about his/her care as much as you would have wanted?

- ❑ I was involved as much as I wanted to be
- ❑ I would have liked to be more involved
- ❑ I would have liked to be less involved
- ❑ Don't know

56. Were any decisions made about his/her care that he/she would not have wanted?

- ❑ No
- ❑ Yes, please specify: ____
- ❑ Don't know

57. Since he/she died, have you talked to anyone from health and supportive services, or from a bereavement service, about your feelings about his/her illness and death?

- ❑ Yes
- ❑ No, I was <u>not</u> aware of these services but I would have <u>liked</u> to use them
- ❑ No, I was <u>not</u> aware of these services but I was <u>not interested</u> anyway
- ❑ No, I was aware of these services but I was <u>not interested</u> anyway
- ❑ Not sure

<u>PART G:</u> Advance Care Planning

The following questions pertain to discussions surrounding Advance Care Planning, which is defined as planning for care in case your family member/friend is not able to make decisions about him/herself in the future.

58. Was he/she given the opportunity to discuss **<u>advance care planning</u>** with his/her health care providers?

- ❑ Yes, definitely
- ❑ Yes, to some extent
- ❑ No, he/she was not given the opportunity – **go to Q61**
- ❑ Don't know **– go to Q61**

59. Who was the **<u>main</u>** provider who discussed **<u>advance care planning</u>** with him/her? *Tick one only*

- ❑ His/her family doctor
- ❑ Palliative care doctor
- ❑ Nurse practitioner
- ❑ Oncologist
- ❑ Other doctor/specialist (e.g., cardiologist)
- ❑ Homecare nurse (not nurse practitioner)
- ❑ Hospital nurse
- ❑ Hospice nurse
- ❑ Other, please specify: ________________
- ❑ Don't know

60. Did this discussion come:

- ❑ Too early
- ❑ Too late
- ❑ At the right time
- ❑ Don't know

61. Did your healthcare providers help you, the caregiver, understand what to expect/how to prepare for his/her death?

- ❑ Yes, definitely
- ❑ Yes, to some extent
- ❑ No
- ❑ Don't know

PART H: Final Thoughts You Wish to Share

62. Lastly, please share any other thoughts you have about what was **good** and what was **bad** about the care services that were provided to your family member or friend within the **last 3 months** of life.

What, if anything, was good about the care?

____

____

____

____

____

____

What, if anything, was bad about the care?

____

____

____

____

____

____

Choosing to name a provider can have an effect on your anonymity. All efforts will be made to maintain the highest level of anonymity and information security.

Thank you!

The time you spent completing this survey is greatly appreciated

Please return completed survey in the stamped, self-addressed envelope provided As Soon As Possible.
